# Supplementary material for: Physiotherapy Rehabilitation in Subjects Diagnosed with Subacromial Impingement Syndrome Does Not Normalize Periscapular and Rotator Cuff Muscle Onset Time of Activation
Source: Int J Environ Res Public Health. 2021 Aug 25;18(17):8952. doi: 10.3390/ijerph18178952 (PMC8430487; doi:10.3390/ijerph18178952)
Supplement: Supplementary file 1 [file ijerph-18-08952-s001.zip › ijerph-1301767-supplementary.pdf]

International Journal of
Environmental Research and Public Health

MDPI

Supplementary Materials:

Table S1. Descriptive statistics of EMG onset time of activation (ms) during loaded flexion movement prior and after standardized physiotherapy treatment for SIS movement at slow, medium and fast speed, and loaded and unloaded condition.

Muscle/ Speed	Condition	n	Median	IQR	95% CI Sup Lim	95% CI Inf Lim	P-Value	Hedges
			FLEXION MOVEMENT LOAD					
TS/Slow	Pre	23	73.4	418.5	243.6	−96.7	**0.01**	2.0
	Post	22	−639.5	312.5				
DA/Slow	Pre	26	−216.2	431.9	−51.0	−381.3	**0.00**	0.3
	Post	22	−325.9	357.7				
DM/Slow	Pre	23	38.7	456.5	224.3	−147.0	0.07	−0.3
	Post	22	214.6	633.1				
DP/Slow	Pre	11	−67.8	748.4	372.2	−507.8	0.68	0.4
	Post	19	−211.5	310.8				
TI/Slow	Pre	18	−45.2	737.9	293.9	−384.3	0.72	0.2
	Post	21	−115.9	178.0				
SA/Slow	Pre	16	−288.0	107.5	236.1	−812.2	**0.05**	−0.7
	Post	12	95.4	534.3				
Subsc/Slow	Pre	8	−66.0	449.6	243.9	−376.0	0.11	0.0
	Post	13	−41.6	527.4				
Pec/Slow	Pre	14	42.1	508.2	307.0	−222.7	0.79	0.5
	Post	12	−170.2	365.6				
SS/Slow	Pre	3	14.5	431.7	500.5	−471.6	0.18	0.4
	Post	8	−118.1	381.1				
IF/Slow	Pre	6	−574.2	573.6	−117.6	−103.8		−0.7
	Post	6	−304.1	375.7				
TS/M	Pre	29	−333.2	392.0	−191.3	−475.2	0.26	0.1
	Post	22	−353.2	188.3				
DA/M	Pre	28	−574.9	317.5	−457.9	−691.9	0.21	−0.4
	Post	21	−454.8	357.8				
DM/M	Pre	29	−62.9	366.2	69.7	−195.5	**0.00**	−0.5
	Post	20	149.0	560.6				
DP/M	Pre	19	235.6	383.3	407.1	64.1	**0.02**	0.6
	Post	17	13.0	381.3				
TI/M	Pre	27	−27.8	512.2	164.4	−220.0	**0.02**	0.4
	Post	10	−168.5	408.4				
SA/M	Pre	23	−86.8	546.5	135.4	−309.0	0.65	0.1
	Post	18	−125.5	394.4				
Pec/M	Pre	27	−254.9	481.1	−74.4	−435.4	**0.05**	−0.6
	Post	17	21.5	538.6				
Subsc/M	Pre	10	−140.5	559.7	204.6	−485.6	0.11	−0.4
	Post	14	22.1	419.7				
SS/M	Pre	9	145.4	415.5	415.4	−124.7	0.22	0.4
	Post	10	−39.9	577.0				
IF/M	Pre	7	280.8	480.3	634.8	−73.1		1.9
	Post	8	−294.3	346.6				
TS/Fast	Pre	30	−233.7	281.7	−133.4	−334.0	0.59	−0.1
	Post	21	−192.5	478.2				
DA/Fast	Pre	30	−315.7	274.9	−217.8	−413.6	0.59	0.2
	Post	9	−404.1	522.7				
DM/Fast	Pre	30	−45.3	256.4	46.0	−136.6	0.20	0.9
	Post	22	−379.3	137.6				
DP/Fast	Pre	26	283.6	361.0	421.6	145.5	0.53	0.9
	Post	17	−122.0	456.8				
TI/Fast	Pre	30	−120.0	398.4	21.8	−261.9	0.69	−0.5
	Post	16	158.1	497.2				
SA/Fast	Pre	26	−166.0	264.4	−64.9	−267.1	0.86	0.1
	Post	19	−219.1	400.6	−39.9	−398.3		
Pec/Fast	Pre	28	−202.3	425.1	−45.6	−358.9	0.05	−0.4

	Post	19	−24.3	369.7				
Subsc/Fast	Pre	13	54.5	507.6	329.0	−220.0	0.59	0.8
	Post	6	−338.0	408.6				
SS/Fast	Pre	15	414.1	398.5	614.7	213.5	0.14	2.0
	Post	7	−399.0	228.5				
IF/Fast	Pre	7	422.5	287.6	634.5	210.5		1.5
	Post	8	−255.9	248.3				

Median, IQR (Standard deviation), p> 0.05, Effect size Hedges 's, 95% Confidence Interval (CI) Superior Limit (Sup Lim) and Inferior Limit (Inf Lim), and p-value of comparative EMG onset time of activation of flexion movement in different speeds (slow, medium, fast) and load (loaded and unloaded). Non parametric data. UT (Upper Trapezius), AD (Anterior Deltoid), MD (Mid Deltoid), PD (Posterior Deltoid), LT (Lower Trapezius), SA (Serratus Anterior), Pec (Pectoralis Major), Subsc (Subscapularis), SS (Supraspinatus), IF (infraspinatus). Slow (Slow speed), M (Medium Speed), Fast (Fast Speed).

Table S2. Descriptive statistics of EMG onset time of activation (ms) during unloaded flexion movement prior and after standardized physiotherapy treatment for SIS movement at slow, medium and fast speed, and loaded and unloaded condition.

FLEXION MOVEMENT UNLOAD								
**Muscle/ Speed**	**Condition**	**n**	**Median**	**IQR**	**95% CI Sup Lim**	**95% CI Inf Lim**	**P-Value**	**Hedges**
TS/Slow	Pre	28	−227.7	534.4	−30.7	−424.6	0.37	3.08
	Post	22	−422.2	192.1				
DA/Slow	Pre	29	−657.1	416.6	−506.2	−808.0	**0.00**	3.11
	Post	22	−433.6	176.2				
DM/Slow	Pre	30	−126.3	471.7	41.6	−294.3	0.53	0.35
	Post	22	−271.3	260.3				
DP/Slow	Pre	16	113.3	586.1	399.1	−172.4	0.33	0.62
	Post	19	−172.3	349.4				
TI/Slow	Pre	24	−69.8	588.5	164.5	−304.0	0.58	0.92
	Post	24	34.0	432.0				
SA/Slow	Pre	23	−205.5	615.0	44.6	−455.5	0.43	0.16
	Post	17	−104.4	497.6				
Subsc/Slow	Pre	13	−691.0	380.6	−485.1	−896.8	0.07	5.75
	Post	14	18.4	500.1				
Pec/Slow	Pre	24	−207.0	544.9	9.9	−423.9		
	Post	13	−215.6	425.8			0.29	0.79
SS/Slow	Pre	10	−799.5	261.5	−638.3	−960.7	0.18	0.72
	Post	9	−204.0	466.8				
IF/Slow	Pre	8	−315.4	638.9	125.1	−755.9		2.85
	Post	7	−71.3	341.6				
TS/M	Pre	29	−478.3	409.1	−330.2	−626.4	**0.00**	3.38
	Post	22	156.4	355.6				
DA/M	Pre	29	−761.2	287.7	−657.0	−865.4	**0.00**	5.94
	Post	17	80.3	506.6				
DM/M	Pre	30	−350.7	281.4	−250.5	−450.9	0.44	0.40
	Post	9	−385.0	582.8				
DP/M	Pre	19	63.1	457.7	267.8	−141.7	0.44	2.73
	Post	17	−49.3	482.8				
TI/M	Pre	28	−251.7	587.4	−35.3	−468.2	0.65	0.16
	Post	19	−100.7	507.3				
SA/M	Pre	27	−205.7	557.8	3.6	−415.1	0.88	0.47
	Post	22	−255.3	475.8				
Pec/M	Pre	29	−383.9	571.5	−176.9	−590.8		
	Post	20	−282.6	502.6			0.46	0.02
Subsc/M	Pre	12	−555.9	462.0	−295.8	−815.9	0.40	1.43
	Post	12	−80.6	474.8				
SS/M	Pre	14	−89.1	674.5	262.5	−440.6	0.59	3.59
	Post	8	−166.5	359.4				
IF/M	Pre	9	−236.2	537.6	113.2	−585.6		0.07
	Post	9	94.8	441.6				
TS/Fast	Pre	29	−324.8	367.3	−191.9	−457.8	**0.00**	4.62
	Post	19	220.5	443.0				

DA/Fast	Pre	30	−431.2	314.5	−319.3	−543.2	0.09	0.68
	Post	22	−595.0	175.7				
DM/Fast	Pre	30	−224.0	321.7	−109.5	−338.6	0.39	0.14
	Post	21	−299.4	194.0				
DP/Fast	Pre	24	202.4	362.7	346.8	58.0	**0.05**	2.00
	Post	20	−133.5	345.3				
TI/Fast	Pre	29	−174.6	397.4	−30.7	−318.5	0.72	0.48
	Post	20	−56.6	451.5				
SA/Fast	Pre	28	−248.3	373.0	−110.8	−385.7	0.90	0.06
	Post	21	−228.9	335.3				
Pec/Fast	Pre	28	−300.3	447.8	−135.3	−465.3		
	Post	21	−161.6	220.5			0.75	0.12
Subsc/Fast	Pre	14	−293.0	609.7	24.7	−610.7	0.18	1.15
	Post	9	−250.8	413.6				
SS/Fast	Pre	13	−79.3	591.7	240.7	−399.3	0.65	2.60
	Post	6	−420.1	305.0				
IF/Fast	Pre	13	−332.6	562.2	−28.5	−636.6		0.18
	Post	8	−222.4	246.8				

Median, IQR (Standard deviation), p> 0.05, Effect size Hedges ‘s, 95% Confidence Interval (CI) Superior Limit (Sup Lim) and Inferior Limit (Inf Lim), and p-value of comparative EMG onset time of activation of flexion movement in different speeds (slow, medium, fast) and load (loaded and unloaded). Non parametric data. UT (Upper Trapezius), AD (Anterior Deltoid), MD (Mid Deltoid), PD (Posterior Deltoid), LT (Lower Trapezius), SA (Serratus Anterior), Pec (Pectoralis Major), Subsc (Subscapularis), SS (Supraspinatus), IF (infraspinatus). Slow (Slow speed), M (Medium Speed), Fast (Fast Speed).

Table S3. Descriptive statistics of EMG onset time of activation (ms) during loaded scaption movement prior and after standardized physiotherapy treatment for SIS movement at slow, medium, and fast speed, and loaded and unloaded condition.

**SCAPTION MOVEMENT LOAD**								
**Muscle/ Speed**	**LOAD**							
	**Condition**	**n**	**Median**	**IQR**	**95% CI Sup Lim**	**95% CI Inf Lim**	**P-Value**	**Hedges**
TS/Slow	Pre	24	−274.7	486.0	−81.2	−468.1	**0.03**	0.6
	Post	18	−500.6	160.2				
DA/Slow	Pre	27	−299.3	441.7	−133.6	−465.1	**0.00**	0.0
	Post	22	−309.4	121.3				
DM/Slow	Pre	26	−23.7	469.6	155.9	−203.3	**0.00**	0.2
	Post	22	−92.9	248.0				
DP/Slow	Pre	17	173.6	506.0	412.9	−65.7	**0.05**	0.7
	Post	20	−126.9	499.1				
TI/Slow	Pre	22	−220.1	531.2	0.8	−440.9	0.66	−0.2
	Post	20	−119.5	386.6				
SA/Slow	Pre	17	−177.3	500.7			0.75	−0.2
	Post	15	−75.9	555.1	203.6	−355.4		
Subsc/Slow	Pre	16	−35.3	537.0	226.5	−297.2	0.12	0.3
	Post	13	−151.4	390.1				
Pec/Slow	Pre	9	−130.4	587.6	251.5	−512.4	0.35	0.4
	Post	11	−310.1	408.6				
SS/Slow	Pre	9	139.7	478.8	450.9	−171.5	0.65	0.3
	Post	9	−8.8	359.5				
IF/Slow	Pre	4	−92.8	659.3	550.1	−735.6		0.3
	Post	7	−197.3	276.3				
TS/M	Pre	28	−357.3	323.0	−238.3	−476.3	0.10	−0.8
	Post	22	−133.4	209.0				
DA/M	Pre	30	−499.4	313.0	−388.0	−610.9	0.99	−1.1
	Post	22	−111.5	334.2				
DM/M	Pre	29	−250.2	365.7	−117.7	−382.6	**0.00**	−1.3
	Post	22	312.9	502.7				
DP/M	Pre	23	230.8	414.0	399.2	62.5	**0.00**	0.6
	Post	8	−5.1	435.7				
TI/M	Pre	23	−204.5	493.6	−3.8	−405.2	0.72	0.0
	Post	23	−2245.0	297.6				
SA/M	Pre	26	−2.6	557.4			0.30	0.6

	Post	17	−235.0	358.7	−65.3	−404.6		
Pec/M	Pre	21	114.6	468.5	314.0	−84.8	0.23	0.4
	Post	19	−70.2	493.7				
Subsc/M	Pre	9	−168.7	624.1	236.9	−574.3	0.72	−0.4
	Post	10	19.1	519.6				
SS/M	Pre	8	−306.8	661.5	149.3	−762.9		−0.9
	Post	6	−15.2	395.0				
IF/M	Pre	10	106.4	536.9	437.5	−224.7	0.18	0.2
	Post	10	3.7	520.8				
TS/Fast	Pre	29	−164.5	361.8	−33.5	−295.5	0.64	0.2
	Post	22	−218.0	222.4				
DA/Fast	Pre	30	−163.7	380.5	−28.2	−299.2	0.89	1.1
	Post	22	−522.8	175.8				
DM/Fast	Pre	29	−114.6	387.3	25.6	−254.8	0.91	1.1
	Post	21	−546.8	363.2				
DP/Fast	Pre	29	188.9	413.5	338.6	39.2	0.83	0.3
	Post	19	20.4	431.2				
TI/Fast	Pre	25	−88.2	425.3	77.7	−254.0	1.00	0.0
	Post	25	−88.2	425.3				
SA/Fast	Pre	25	88.8	449.6			1.00	0.1
	Post	19	18.6	480.0	233.4	−196.1		
Pec/Fast	Pre	28	135.0	386.4	277.4	−7.3	0.35	0.0
	Post	19	121.3	495.9				
Subsc/Fast	Pre	13	−113.7	600.7	211.2	−438.6	0.65	0.3
	Post	10	−287.6	496.9				
SS/Fast	Pre	13	179.8	361.3	375.3	−15.6	0.08	0.8
	Post	6	−248.8	253.5				
IF/Fast	Pre	8	462.0	317.7	681.0	242.9		1.9
	Post	7	−351.0	311.6				

Median, IQR (Standard deviation), p> 0.05, Effect size Hedges ′s, 95% Confidence Interval (CI) Superior Limit (Sup Lim) and Inferior Limit (Inf Lim), and p-value of comparative EMG onset time of activation of flexion movement in different speeds (slow, medium, fast) and load (loaded and unloaded). Non parametric data. UT (Upper Trapezius), AD (Anterior Deltoid), MD (Mid Deltoid), PD (Posterior Deltoid), LT (Lower Trapezius), SA (Serratus Anterior), Pec (Pectoralis Major), Subsc (Subscapularis), SS (Supraspinatus), IF (infraspinatus). Slow (Slow speed), M (Medium Speed), Fast (Fast Speed)..

Table S4. Descriptive statistics of EMG onset time of activation (ms) during unloaded scaption movement prior and after standardized physiotherapy treatment for SIS movement at slow, medium, and fast speed, and loaded and unloaded condition.

SCAPTION MOVEMENT UNLOAD								
**Muscle/ Speed**	**LOAD**							
	**Condition**	**n**	**Median**	**IQR**	**95% CI Sup Lim**	**95% CI Inf Lim**	**P-Value**	**Hedges**
TS/Slow	Pre	29	−350.1	450.5	−186.9	−513.2	0.74	0.31
	Post	22	−475.1	132.1				
DA/Slow	Pre	30	−719.1	291.9	−615.2	−823.0	0.77	5.40
	Post	20	−189.7	196.3				
DM/Slow	Pre	29	−348.7	415.5	−198.3	−499.1	**0.01**	5.85
	Post	19	67.4	360.3				
DP/Slow	Pre	22	222.3	531.5	443.3	1.3	0.11	0.48
	Post	16	−80.2	368.0				
TI/Slow	Pre	24	−187.1	561.3	36.3	−410.6	0.88	0.39
	Post	15	−138.7	511.9				
SA/Slow	Pre	26	−219.9	548.1	−10.3	−429.5	0.46	0.05
	Post	15	−252.7	491.3				
Subsc/Slow	Pre	24	−38.1	533.1	174.1	−250.2		
	Post	18	−94.9	597.4			0.41	0.41
Pec/Slow	Pre	11	−469.4	425.6	−219.2	−719.6	0.89	0.46
	Post	10	−73.6	514.1				
SS/Slow	Pre	4	−58.1	593.0	520.1	−636.2	0.89	7.59
	Post	10	11.8	550.5				
IF/Slow	Pre	8	−251.7	779.1	285.5	−788.8		1.27

	Post	3	−287.7	145.4				
TS/M	Pre	27	−559.9	375.7	−418.9	−700.9	**0.00**	1.41
	Post	21	−135.0	194.3				
DA/M	Pre	30	−698.7	265.2	−604.3	−793.1	**0.00**	2.24
	Post	22	−197.0	146.7				
DM/M	Pre	29	−513.7	362.7	−382.3	−645.0	0.19	0.64
	Post	22	−414.4	317.7				
DP/M	Pre	24	93.2	461.9	277.0	−90.7	0.72	0.85
	Post	8	−5.1	435.7				
TI/M	Pre	26	−249.8	533.5	−45.8	−453.8	0.33	0.50
	Post	18	−116.6	393.8				
SA/M	Pre	28	−284.6	507.3	−97.7	−471.6	0.16	0.47
	Post	18	−307.9	408.2				
Pec/M	Pre	27	−210.9	510.7	−19.2	−402.5		
	Post	20	−228.2	505.0			0.51	0.03
Subsc/M	Pre	13	−173.5	698.0	204.0	−551.0	0.87	0.23
	Post	10	70.3	439.0				
SS/M	Pre	8	88.3	663.8	546.0	−369.4		0.53
	Post	0						
IF/M	Pre	10	−325.8	644.8	71.8	−723.4		0.09
	Post	8	−89.0	409.1				
TS/Fast	Pre	29	−368.3	288.3	−263.9	−472.6	**0.00**	5.22
	Post	20	282.1	451.2				
DA/Fast	Pre	29	−493.8	316.4	−379.3	−608.4	0.52	8.06
	Post	20	80.0	407.4				
DM/Fast	Pre	29	−373.1	306.6	−262.1	−484.1	**0.05**	4.94
	Post	22	−68.7	487.4				
DP/Fast	Pre	27	−158.6	365.7	−21.4	−295.8	0.66	1.66
	Post	19	20.4	431.2				
TI/Fast	Pre	28	−265.2	434.5	−105.1	−425.4	0.07	1.35
	Post	17	−101.0	294.5				
SA/Fast	Pre	28	−164.5	447.5	0.4	−329.4	**0.02**	0.42
	Post	19	−137.3	410.1				
Pec/Fast	Pre	27	−191.6	418.2	−34.6	−348.5		
	Post	19	−93.7	383.8			0.86	0.73
Subsc/Fast	Pre	19	−8.2	510.5	220.1	−236.6	0.69	0.16
	Post	5	74.2	317.3				
SS/Fast	Pre	15	121.8	528.3	387.8	−144.2		0.01
	Post	7	−526.4	284.6				
IF/Fast	Pre	13	−97.1	628.0				8.25
	Post	0						

Median, IQR (Standard deviation), p> 0.05, Effect size Hedges 's, 95% Confidence Interval (CI) Superior Limit (Sup Lim) and Inferior Limit (Inf Lim), and p-value of comparative EMG onset time of activation of flexion movement in different speeds (slow, medium, fast) and load (loaded and unloaded). Non parametric data. UT (Upper Trapezius), AD (Anterior Deltoid), MD (Mid Deltoid), PD (Posterior Deltoid), LT (Lower Trapezius), SA (Serratus Anterior), Pec (Pectoralis Major), Subsc (Subscapularis), SS (Supraspinatus), IF (infraspinatus). Slow (Slow speed), M (Medium Speed), Fast (Fast Speed)..

Table S5. Descriptive statistics of EMG onset time of activation (ms) during loaded abduction movement prior and after standardized physiotherapy treatment for SIS movement at slow, medium, and fast speed, and loaded and unloaded condition.

ABDUCTION MOVEMENT LOAD								
	LOAD							
**Muscle/ Speed**	**Condition**	**n**	**Median**	**IQR**	**95% CI Sup Lim**	**95% CI Inf Lim**	**P-Value**	**Hedges**
TS/Slow	Pre	26	−100.1	475.1	81.6	−281.8	0.18	−0.7
	Post	21	201.2	333.9				
DA/Slow	Pre	25	−250.6	415.0	−196.3	−528.4	**0.01**	0.0
	Post	21	−177.9	320.8				
DM/Slow	Pre	25	−362.3	425.7	−196.3	−528.4	**0.01**	0.0
	Post	21	−370.0	208.7				
DP/Slow	Pre	24	−82.5	598.5	155.8	−320.7	0.98	−0.2

	Post	19	20.4	431.2				
TI/Slow	Pre	16	−222.3	647.9	93.6	−538.1	0.92	0.0
	Post	16	−222.3	647.9				
SA/Slow	Pre	18	80.4	554.1			0.22	0.6
	Post	17	−212.7	531.7	38.8	−464.1		
Subsc/Slow	Pre	9	−417.0	475.9	−107.6	−726.3	0.13	−0.9
	Post	14	27.5	519.4				
Pec/Slow	Pre	8	−376.0	545.4	0.0	−752.0	0.11	−0.3
	Post	10	−205.5	492.6				
SS/Slow	Pre	11	−443.1	664.6	−52.4	−833.9	0.11	−0.6
	Post	9	−179.6	445.1				
IF/Slow	Pre	6	−504.2	551.0	−65.5	−942.8		−0.3
	Post	6	−341.2	476.5				
TS/M	Pre	25	−265.0	354.6	−126.7	−403.3	0.44	0.5
	Post	11	−439.5	467.7				
DA/M	Pre	28	−432.6	338.0	−308.1	−557.2	0.09	−1.2
	Post	22	−82.7	270.2				
DM/M	Pre	28	−432.6	338.0	−308.1	−557.2	0.09	−1.2
	Post	22	−82.7	270.2				
DP/M	Pre	26	−226.3	432.4	−61.0	−391.7	**0.05**	−0.7
	Post	19	28.3	416.7				
TI/M	Pre	22	−82.7	580.9	158.9	−324.2	**0.01**	0.0
	Post	22	−82.7	580.9				
SA/M	Pre	25	−36.8	477.7			0.42	0.3
	Post	16	−138.7	379.0	46.0	−323.5		
Pec/M	Pre	19	102.6	476.4	315.8	−110.5	0.06	0.4
	Post	19	−72.1	479.8				
Subsc/M	Pre	11	−151.1	589.2	195.3	−497.5	1.00	−0.1
	Post	12	−105.5	388.3				
SS/M	Pre	11	−205.1	457.5	63.9	−474.1		0.1
	Post	9	−251.4	368.5				
IF/M	Pre	6	−322.9	770.4	290.4	−936.2		−0.7
	Post	9	39.9	325.4				
TS/Fast	Pre	28	−228.2	282.5	−124.1	−332.3	0.28	−0.6
	Post	22	−15.3	438.0				
DA/Fast	Pre	29	−216.5	324.1	−99.2	−333.9	**0.04**	0.0
	Post	19	−222.1	308.6				
DM/Fast	Pre	29	−216.5	324.1	−99.2	−333.9	**0.04**	0.0
	Post	19	−222.1	308.6				
DP/Fast	Pre	27	−123.3	369.4	15.4	−261.9	0.93	−0.3
	Post	15	48.2	512.3				
TI/Fast	Pre	26	−87.3	531.7	116.1	−290.6	0.17	0.0
	Post	26	−87.3	531.7				
SA/Fast	Pre	26	14.8	384.7			0.88	0.2
	Post	18	−105.7	490.4	119.7	−331.1		
Pec/Fast	Pre	25	270.2	447.0	444.6	95.9	0.50	0.5
	Post	20	−2.1	455.8				
Subsc/Fast	Pre	11	41.7	437.4	298.9	−215.4	0.59	0.7
	Post	8	−240.3	438.1				
SS/Fast	Pre	14	177.2	314.0	340.9	13.6		3.0
	Post	6	−552.6	228.8				
IF/Fast	Pre	11	−152.7	731.7	277.6	−582.9	0.35	0.2
	Post	7	−244.0	520.7				

Median, IQR (Standard deviation), p> 0.05, Effect size Hedges 's, 95% Confidence Interval (CI) Superior Limit (Sup Lim) and Inferior Limit (Inf Lim), and p-value of comparative EMG onset time of activation of flexion movement in different speeds (slow, medium, fast) and load (loaded and unloaded). Non parametric data. UT (Upper Trapezius), AD (Anterior Deltoid), MD (Mid Deltoid), PD (Posterior Deltoid), LT (Lower Trapezius), SA (Serratus Anterior), Pec (Pectoralis Major), Subsc (Subscapularis), SS (Supraspinatus), IF (infraspinatus). Slow (Slow speed), M (Medium Speed), Fast (Fast Speed).

Table S6. Descriptive statistics of EMG onset time of activation (ms) during unloaded abduction movement prior and after standardized physiotherapy treatment for SIS movement at slow, medium, and fast speed, and loaded and unloaded condition.

ABDUCTION MOVEMENT UNLOAD								
Muscle/ Speed	LOAD							
	Condition	n	Median	IQR	95% CI Sup Lim	95% CI Inf Lim	P-Value	Hedges
TS/Slow	Pre	27	−364.4	527.1	−166.6	−562.2	**0.02**	0.38
	Post	19	−601.2	435.4				
DA/Slow	Pre	29	−565.2	381.1	−427.2	−703.2	**0.02**	0.87
	Post	20	−465.5	169.4				
DM/Slow	Pre	29	−565.2	381.1	−427.2	−703.2	**0.02**	0.87
	Post	20	−465.5	169.4				
DP/Slow	Pre	26	−250.4	496.7	−60.5	−440.3	0.69	2.33
	Post	19	28.3	416.7				
TI/Slow	Pre	21	9.4	558.6	247.0	−228.3	1.00	1.56
	Post	20	−210.8	382.4	−44.0	−377.5		
SA/Slow	Pre	24	−257.3	506.2	−55.8	−458.8	0.39	0.75
	Post	15	−69.2	530.4				
Subsc/Slow	Pre	12	37.4	547.7	345.7	−270.9		
	Post	17	−116.4	499.7			0.27	1.00
Pec/Slow	Pre	13	20.2	518.5	300.6	−260.2	0.60	1.08
	Post	10	−152.3	481.6				
SS/Slow	Pre	8	−279.6	569.0	112.6	−671.9	0.65	8.02
	Post	8	−227.5	382.5				
IF/Slow	Pre	6	−546.5	558.8				3.05
	Post	0						
TS/M	Pre	25	−529.9	339.4	−397.6	−662.3	**0.04**	0.01
	Post	22	−248.3	467.2				
DA/M	Pre	27	−620.9	329.5	−497.2	−744.5	**0.01**	1.37
	Post	11	56.0	609.2				
DM/M	Pre	27	−620.9	329.5	−497.2	−744.5	**0.01**	1.37
	Post	11	56.0	609.2				
DP/M	Pre	27	−283.9	439.4	−119.0	−448.8	**0.02**	0.63
	Post	15	48.2	512.3				
TI/M	Pre	25	−128.2	659.8			0.36	0.06
	Post	18	−353.1	390.5	−173.7	−532.6		
SA/M	Pre	26	−232.6	515.2	−35.6	−429.7	0.30	0.08
	Post	17	−268.4	387.1				
Pec/M	Pre	20	46.0	646.9	328.1	−236.1		
	Post	20	−145.7	473.6			0.29	0.16
Subsc/M	Pre	13	−48.9	638.9	296.6	−394.4	0.59	0.06
	Post	10	−63.3	570.7				
SS/M	Pre	10	−53.6	568.6	297.1	−404.2		4.11
	Post	11	−59.2	410.6				
IF/M	Pre	7	−277.4	747.2				0.64
	Post	0						
TS/Fast	Pre	29	−255.2	360.5	−124.7	−385.8	0.43	11.62
	Post	21	−133.0	204.3				
DA/Fast	Pre	28	−256.1	290.7	−148.9	−363.2	0.69	1.48
	Post	20	−221.6	333.5				
DM/Fast	Pre	28	−256.1	290.7	−148.9	−363.2	0.69	1.48
	Post	20	−221.6	333.5				
DP/Fast	Pre	26	−138.0	350.4	−4.0	−272.0	0.57	0.21
	Post	26	−138.0	350.4				
TI/Fast	Pre	27	−168.0	456.1			0.92	1.14
	Post	19	−194.6	252.0	−81.9	−307.4		
SA/Fast	Pre	26	38.3	410.8	195.4	−118.8	**0.03**	0.81
	Post	19	−149.7	407.8				
Pec/Fast	Pre	24	95.8	551.0	315.1	−123.5		

	Post	20	37.3	355.1				
Subsc/Fast	Pre	13	−91.7	462.8	158.5	−342.0	0.72	0.03
	Post	8	−317.7	373.6				
SS/Fast	Pre	14	110.2	584.3	414.7	−194.3		1.19
	Post	6	−427.7	321.9				
IF/Fast	Pre	16	−131.0	659.2				
	Post	0						

Median, IQR (Standard deviation), p> 0.05, Effect size Hedges ‘s, 95% Confidence Interval (CI) Superior Limit (Sup Lim) and Inferior Limit (Inf Lim), and p-value of comparative EMG onset time of activation of flexion movement in different speeds (slow, medium, fast) and load (loaded and unloaded). Non parametric data. UT (Upper Trapezius), AD (Anterior Deltoid), MD (Mid Deltoid), PD (Posterior Deltoid), LT (Lower Trapezius), SA (Serratus Anterior), Pec (Pectoralis Major), Subsc (Subscapularis), SS (Supraspinatus), IF (infraspinatus). Slow (Slow speed), M (Medium Speed), Fast (Fast Speed).
